# Ohmic heating dominates and ultrasound modulates aquafaba structure: A combined approach to design stable plant‐based emulsions

**DOI:** 10.1002/jsfa.70743

**Published:** 2026-05-27

**Authors:** Débora Krichanã, Thaís Caroline Buttow Rigolon, Paula Zambe Azevedo, João Paulo de Melo Lins, Gabriel Oliveira Horta, Linamarys Aparecida de Oliveira Paulo, Márcia Cristina Teixeira Ribeiro Vidigal, Taline Amorim Santos, Isis Rodrigues Toledo Renhe, Jaqueline de Araújo Bezerra, Paulo César Stringheta, Evandro Martins, Pedro Henrique Campelo

**Affiliations:** ^1^ LaCBio, Laboratory of Natural Pigments and Bioactives, Food Technology Department Federal University of Viçosa Viçosa Brazil; ^2^ LHMA, Laboratory of Hygiene and Food Microbiology, Food Technology Department Federal University of Viçosa Viçosa Brazil; ^3^ Food Technology Department Federal University of Viçosa Viçosa Brazil; ^4^ EPAMIG – Instituto de Laticínios Cândido Tostes Juiz de Fora Brazil; ^5^ Federal Institute of Education, Science, and Technology of Amazonas Manaus Brazil

**Keywords:** aquafaba, ohmic heating, ultrasound, emerging technologies, protein functionality, plant‐based emulsions

## Abstract

**BACKGROUND:**

This study investigated the individual and combined effects of ohmic heating (OH, 20 and 40 V cm^−1^) and ultrasound (US, 425 and 850 W) on the chemical, physicochemical, and technological properties of chickpea aquafaba (AF) and its emulsions.

**RESULTS:**

Structural analyses demonstrated that OH was the predominant factor modulating protein unfolding, whereas US acted as a secondary modulator, dispersing aggregates and altering the extent of exposure of hydrophobic and sulfhydryl groups. At moderate OH intensity (20 V cm^−1^), combined treatments (particularly 20/425) promoted higher surface hydrophobicity, increased solubility, greater availability of sulfhydryl groups and more negative zeta potential. These modifications translated into improved emulsion properties, with smaller and more homogeneous oil droplets, higher viscosity and consistency indices, stronger gel‐like behavior (*G*′ ≫ *G*″), and enhanced physical stability (TSI < 4). By contrast, high OH intensity (40 V.cm^−1^), especially when combined with high‐power US (40/850), induced excessive aggregation, reduced protein solubility and impaired interfacial coverage, and also led to unstable emulsions. Confocal microscopy confirmed that moderate treatments produced dense interfacial protein films, whereas severe conditions resulted in flocculation and coalescence.

**CONCLUSION:**

Overall, results highlight that OH drives protein modifications, whereas US fine‐tunes structural rearrangements, and that controlled intensities are essential to enhance AF functionality in plant‐based emulsions. © 2026 The Author(s). *Journal of the Science of Food and Agriculture* published by John Wiley & Sons Ltd on behalf of Society of Chemical Industry.

## INTRODUCTION

The growing demand for new plant‐based food ingredients has led research groups to intensify their efforts to evaluate the techno‐functional and sensory properties of these emerging materials.^1^ In 2012, the total number of publications on plant‐based products did not exceed 70; by 2022, this number had increased to 921 articles, and it continues to rise each year.^2^

Chickpea aquafaba is a byproduct widely used in the development of plant‐based foods, serving as an egg substitute in products such as merengues,^3^ cakes and cookies,^4^ or as a replacement for milk proteins in mayonnaise,^5^ ice creams and puddings.^6^ This versatility is primarily attributed to its emulsifying^7^ and foaming^8^ properties. However, its low protein concentration, compositional heterogeneity and the susceptibility of its proteins to physicochemical stress hinder the formation of cohesive interfacial structures that are resistant to collapse. As a result, products made with aquafaba tend to exhibit phase separation, drainage, and structural instability during storage or processing.^9^, ^10^ Although the chemical composition of aquafaba is complex, with more than 63% (dry basis) consisting of carbohydrates,^11^ proteins appear to play a key role in its technological properties. Consequently, they are often used as a reference parameter for assessing new technologies aimed at modifying aquafaba's functional characteristics.^12^, ^13^, ^14^, ^15^, ^16^

Emerging technologies such as ultrasound (US) and ohmic heating (OH) have been widely explored in food science as efficient strategies to modulate the structural and interfacial properties of biopolymers, particularly proteins.^14^, ^17^, ^18^ OH provides rapid and uniform volumetric heating, promoting controlled denaturation and the exposure of functional groups, such as hydrophobic regions and sulfhydryl groups, which enhance adsorption at the oil–water interface and the formation of more cohesive interfacial films.^19^, ^20^ By contrast, US acts mainly through acoustic cavitation, generating shear forces, microjets and shock waves that reduce aggregate size, improve dispersibility and modulate molecular flexibility.^21^, ^22^ When applied in combination, these technologies exhibit a synergistic effect: OH acts as the primary driver of conformational modifications, whereas US fine‐tunes the aggregation state and structural organization of proteins. This combined approach has proven particularly promising for emulsions, leading to smaller droplet sizes, greater homogeneity, increased viscosity and improved physical stability because of the formation of denser and more highly charged interfacial layers. Moreover, controlling process intensities helps prevent excessive aggregation and loss of functionality, which is essential for designing stable colloidal systems with tailored properties for plant‐based food applications.

To enhance the techno‐functional properties of chickpea aquafaba proteins, our recent study investigated the effects of ohmic heating (OH) on protein structural modulation for meringue production.^14^ The application of a moderate electric field intensity (20 V cm^−1^) significantly improved aquafaba performance, increasing protein solubility by approximately 14%, enhancing free sulfhydryl (–SH) content by 61% and improving viscoelastic moduli (*G*′ and *G*″) by more than 35%, which resulted in denser and more elastic foams. Structural analysis revealed a reduction in *α*‐helix and *β*‐sheet contents and an increase in *β*‐turns, indicating partial unfolding and rearrangement of protein conformations that enhanced interfacial and rheological properties. Consequently, meringues prepared from OH‐treated aquafaba exhibited markedly improved texture and microstructure, reflecting greater structural integrity and foam stability. By contrast, higher electric field intensities (≥ 40 V cm^−1^) caused excessive protein denaturation and aggregation, leading to a 47% reduction in solubility, increased porosity and mechanical weakening of the meringues. Despite these promising results, the OH process has a limited operational window, and its combination with other emerging technologies may provide synergistic effects and improved process control. Treatments such as cold plasma,^23^ ultrasound^18^, ^24^ and pulsed electric fields (PEF)^25^ have been reported to induce conformational changes in proteins, increasing the exposure of hydrophobic groups and disulfide bonds, thereby promoting stronger intermolecular interactions and greater stability of dispersed systems. For example, integrating OH with ultrasound‐assisted processing can promote controlled protein unfolding and dispersion, reducing aggregation at the same time as enhancing solubility and interfacial activity. Alizadeh and Aliakbarlu^26^ demonstrated that whey protein hydrolysates treated with combined OH and ultrasound exhibited approximately 18% higher antioxidant activity than samples treated with either technology individually. Moreover, the combination of PEF and ultrasound proved effective in increasing chickpea protein yield and improving its functional properties, such as solubility and emulsifying capacity.^27^ These findings highlight that integrating OH with complementary emerging technologies may overcome its current limitations, offering greater molecular control, higher energy efficiency and enhanced functionalization of plant‐based proteins for clean‐label food formulations.

The present study aimed to evaluate the combined effects of OH and US on the chemical and techno‐functional properties of pure chickpea aquafaba and aquafaba‐based mayonnaise‐type emulsions. Initially, the effects of electric field intensity (20 and 40 V cm^−1^) and ultrasound power (425 and 850 W), applied both individually and in combination, were assessed on the chemical and physicochemical properties of aquafaba. Following this initial analysis, the combined effects of these emerging technologies were investigated on the technological properties of the emulsions, including rheology, stability and confocal microscopy. From an industrial perspective, the combined OH–US approach offers a scalable strategy to tailor protein functionality using short processing times and relatively low energy inputs, aligning with current demands for efficient and sustainable processing.

## MATERIALS AND METHODS

### Aquafaba production

The aquafaba (AF) was obtained following the methodology described by Krichanã *et al*.,^14^ without modification. In total, 100 g of raw chickpeas was weighed and soaked in 330 mL of distilled water at 27 °C for 16 h, allowing the grains to approximately double in size. After soaking, the chickpeas were rinsed three times with distilled water to remove bitter compounds. Subsequently, portions (100 g) were transferred into Erlenmeyer flasks, each covered with 150 mL of distilled water for cooking. The samples were cooked in a water bath at 95 °C for 90 min, followed by cooling to room temperature. Once cooled, the solid fraction (chickpeas) was separated from the liquid fraction (cooking water). The obtained AF was stored under refrigeration until analysis.

### Experimental design

The experimental levels were defined based on previously published results on the application of ultrasound^15^ and ohmic heating^14^ in aquafaba. The selected levels corresponded to those that produced the most significant modifications in the physicochemical properties of aquafaba, aiming to determine whether the combined effects of OH and US could enhance its techno‐functional properties. Individual and combined treatments using these emerging technologies were performed to assess the initial chemical and physicochemical characteristics. For the evaluation of technological properties of emulsions, such as rheology, stability and confocal microscopy, only the combined treatments were considered. Table 1 presents the experimental levels for the combined OH and US treatments.

**Table 1:** Experimental design for the combined application of emerging technologies (ultrasound and ohmic heating) for the modification of aquafaba

Assay	Ohmic heating (electric field/V cm^−1^)	Ultrasound power (W)
Control	0	0
20/425	20	425
20/850	20	850
40/425	40	425
40/850	40	850

#### Ohmic heating modification

The experiments were conducted using an ohmic heater, in which the system consisted of a voltage generator (Tdgc2‐2 2kVA 220 V AC/60 Hz; Variac, Cleveland, OH, USA) that allowed precise temperature control. The system had a volumetric capacity of 50 mL and dimensions of 9 × 12 × 6 cm. This equipment was connected to two titanium electrodes (9 cm between eletrodes) with opposite charges to ensure process efficiency.^14^ Throughout the experiments, the temperature was meticulously monitored using an integrated digital thermometer, and a magnetic stirrer ensured the homogeneity of the aquafaba. The OH process was carried out at two electric field intensities (20 and 40 V cm^−1^) for approximately 25 s each. The experimental levels were determined by initial tests where the aquafaba was not boiled.

#### Ultrasound modification

After the OH treatment, the same 50 mL of aquafaba was subjected to high‐energy probe ultrasound processing (QR850; Ecosonics, Sao Paulo, Brazil) operating at a frequency of 20 kHz, with a probe tip diameter of 0.3 cm, length of 16.5 cm and total power of 850 W. The processing was conducted for 10 min at power levels of 425 and 850 W. The treatment was performed in an ice bath to ensure that the observed modifications in the aquafaba resulted exclusively from the mechanical effects of the ultrasonic waves, rather than from a temperature increase.

### Intrinsic fluorescence

The intrinsic fluorescence of aquafaba was measured on a microplate reader (SpectraMax® i3x; Molecular Devices, Shanghai, China) as described previously,^28^ with few modifications. Briefly, protein samples were diluted to 1 mg mL^−1^ using 10 mm phosphate buffer (pH 7.0) and fluorescence spectra were recorded at an excitation wavelength of 280 nm and an emission wavelength of 300–500 nm.

### Surface hydrophobicity

The surface hydrophobicity of the samples was determined according to the method described by Chelh *et al*.,^29^ with modifications. One milliliter of the suspension was mixed with 200 μL of 1 mg mL^−1^ bromophenol blue (BPB) in distilled water and thoroughly homogenized. The addition of BPB slightly decreased the solution's pH from 6.0 to 5.8. A control consisted of adding 200 μL of 1 mg mL^−1^ BPB (in distilled water) to 1 mL of 20 mm phosphate buffer at pH 6.0. Samples and control were kept under agitation at room temperature for 10 min and then centrifuged for 15 min at 2000 × *g*. The absorbance of the supernatant (diluted 1:10) was measured at 595 nm against a phosphate buffer blank. The amount of bound BPB was calculated using:

(1) $$\mathrm{BPB}\;\text{bound}\;\left(\mathrm{\mu g}\right)=200\;\mathrm{\mu g}\times \frac{{\mathrm{Abs}}_{\text{blank}}-{\mathrm{Abs}}_{\text{sample}}}{{\mathrm{Abs}}_{\text{blank}}}$$

### Free sulfhydryl

The concentration of free sulfhydryl (SH) groups was determined following the methodology proposed by Ellman *et al* ^30^ The samples were dissolved in distilled water to reach a protein concentration of 40 mg mL^−1^. Then, 0.1 mL of the suspension was mixed with 1.9 mL of Tris‐glycine buffer (0.09 m glycine, 0.086 m Tris and 0.004 m EDTA, pH 8.0) containing 2.5% w/v SDS (sodium dodecyl sulfate) to obtain a final protein concentration of 2 mg·mL^−1^. Afterward, 12 μL of Ellman's reagent was added, and the mixture was stirred and stored in the dark for 20 min. Following the incubation period, 200 μL of the solution was transferred to microplates. The absorbance was measured using a microplate reader (SpectraMax® i3x; Molecular Devices) at a wavelength of 412 nm. The blank sample was prepared by replacing the protein solution with buffer solution (pH 8.0) and adding Ellman's reagent. The SH concentration (mmol SH g^−1^ protein) was calculated using:

(2) $$\mathrm{SH}=1000\times \frac{{\mathrm{Abs}}_{\text{sample}}-{\mathrm{Abs}}_{\text{blank}}}{13600}$$

### Solubility

The percentage solubility of AF was determined using the Bradford method.^31^ In summary, 3 g of each protein concentrate was dissolved in 60 mL of distilled water. The pH was adjusted to 7 using 1 m HCl or NaOH solutions. Aliquots of 2 mL were taken and transferred to Eppendorf tubes. The tubes were agitated for 20 min at room temperature (25 °C) and then centrifuged at 10 000 × *g* for 10 min at 4 °C using a centrifuge (Centrifuge 5804 R; Eppendorf, Hamburg, Germany). In total, 200 μL of the supernatant from each tube was pipetted into microplate wells, and absorbance was measured at 595 nm using a microplate reader (SpectraMax® i3x; Molecular Devices).

### Zeta potential

AF solutions (0.01 g.mL^−1^) was diluted 15 times and their charge density (zeta potential) was measured in the Litesizer 500® (Anton Paar GmbH, Graz, Austria).^17^ The pH range of the samples was 5.75–6.14.

### Production of emulsions

For the preparation of emulsions, only the combined treatments and the control (untreated sample) were considered to evaluate the effect of the dual‐technology application on the technological properties of the emulsions. The emulsions were prepared at a 1:3 ratio (aquafaba/soybean oil) and homogenized using a high‐energy homogenizer (T25 Digital ULTRA TURRAX; Ika, Guangzhou, China) at 15 000 rpm for 3 min. The oil phase was gradually added to the aquafaba during the first minute of homogenization. These conditions were established through preliminary tests to ensure that the effects of the structural modifications induced by OH and/or US could be clearly observed.

### Multiple light scattering

The multiple light scattering of the emulsions with different oil phases was measured using a Turbiscan Tower instrument (Formulaction, Toulouse, France). For this, 30 mL of fresh emulsions were monitored in the sample chamber for 24 h (25 °C) to obtain the backscattering (ΔBS) and Turbiscan Stability Index (TSI). The TSI values were automatically calculated using TurbiSoft‐2.0.0.33® software.^32^

### Rheology

The rheological properties of aquafaba emulsions were investigated using a controlled‐stress rheometer (Modular Compact Rheometer – MCR 102e; Anton Paar GmbH) with a parallel plate geometry (50 mm in diameter) at room temperature. The gap in all rheological tests was set to 2 mm. Rheological experiments were performed in triplicate.^14^

#### Steady shear

The flow behavior of the meringue was studied within the shear rate range 0.001–100 s^−1^. The shear stress *versus* shear rate data were fitted to the Power Law model:

(3) $$\tau =k\gamma^{n}$$

where *τ* is the shear stress (Pa), *τ*₀ is the yield stress (Pa), *k* is the consistency index (Pa·s^n^) and *n* is the flow behavior index (dimensionless).

#### Frequency sweep

The frequency dependence of the dynamic viscoelastic moduli (storage modulus *G*′ and loss modulus *G*″) of the aquafaba meringue samples was determined through a frequency sweep test ranging from 0.01 to 60 Hz at a constant strain amplitude of 0.5%, which was within the linear viscoelastic region (LVE) for all samples.^33^

### Confocal laser scanning microscopy

The morphology of the aquafaba emulsions was observed using a LSM 900 microscope (Carl Zeiss GmbH, Oberkochen, Germany). The proteins and the oil phase were fluorescently labelled by adding 5 ppm FITC and 5 ppm Nile Red, respectively (Sigma‐Aldrich, St Louis MO, USA). The excitation wavelengths of Nile red and Rhodamine B in the emulsion fluorescent dye were 488 and 540 nm, respectively.^34^

### Statistical analysis

Data were evaluated based on triplicate repetitions of the experimental analyses. Results were assessed using the mean ± SD and analysis of variance, with Tukey's test applied for comparison, considering a *P* < 0.05 and a confidence level of 95%. Statistical analysis was performed using Jamovi, version 2.4.14 (https://www.jamovi.org).

## RESULTS AND DISCUSSION

### Heating kinetics

**Figure 1:**
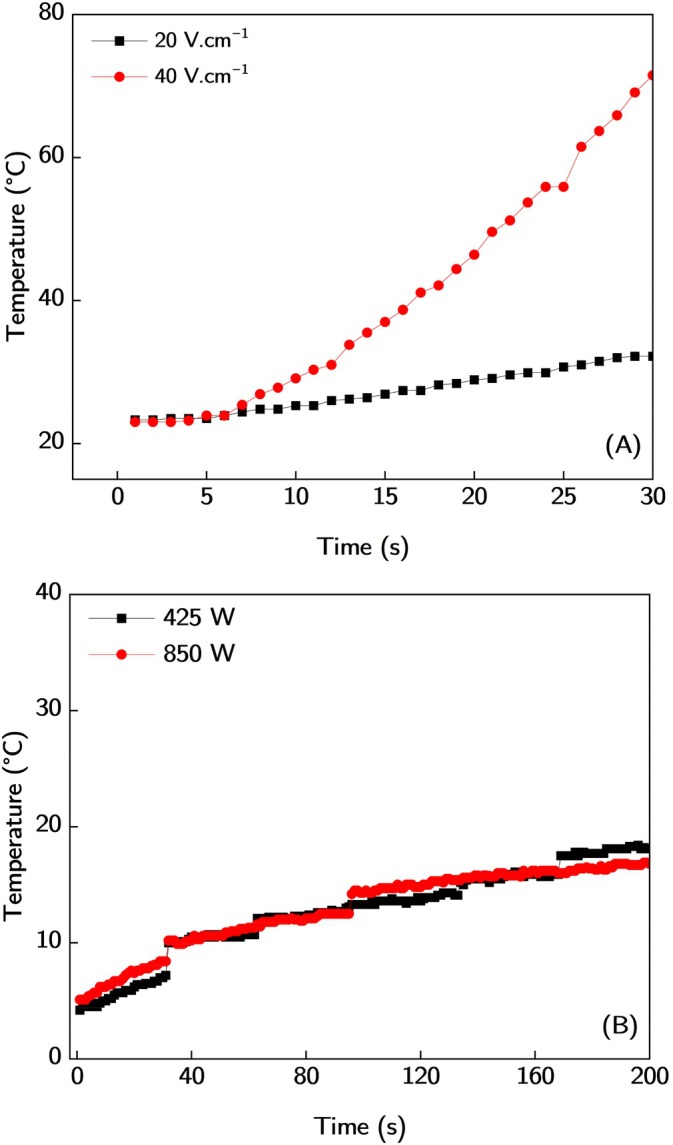
Thermal curves for aquafaba processing by ohmic heating (A) and ultrasound (B).

All types of food processing that involve heat can influence the physicochemical and technological properties of proteins. To determine these effects, the temperature of the aquafaba was monitored throughout the modification process by OH and US (Fig. 1). The electrical parameters of the OH may be consulted in a previous study.^14^ For the samples treated with OH (Fig. 1(A)), it was observed that increasing the electric field intensity was positively correlated with a rise in temperature.^14^ Alsalman *et al*.^35^ reported a complete denaturation temperature range for aquafaba proteins between 109 and 127 °C. Therefore, the temperature range adopted in this study (<80 °C) may not have caused significant alterations in protein structure. Nonetheless, such modifications appeared sufficient to expose certain active sites on the protein molecules capable of interacting with water and/or oil (as addressed in later sections). By contrast, the US treatments showed no temperature differences among the various conditions (Fig. 1(B)). This uniform temperature profile was intentional because the process was carried out in an ice bath to ensure that the structural modifications of the proteins resulted solely from the shear forces, mechanical shock of sound waves, and cavitation phenomena.

### Intrinsic fluorescence

Fluorescence spectroscopy applied to proteins is a non‐invasive tool for investigating structural aspects, allowing the evaluation of how fluorophores behave in different molecular microenvironments, whether polar or non‐polar.^36^

The OH treatments showed antagonistic results: at lower electric field intensity, a substantial increase in relative fluorescence units (RFU) was observed, whereas, at a higher electric field intensity, RFU values decreased below those of the control aquafaba. These findings are consistent with the results of Wang *et al*.,^37^ who reported that, when the electric field intensity in OH increased, the structure of soy proteins expanded, causing tryptophan residues to come into contact with the aqueous solution, thereby quenching fluorescence emission.

By contrast to the OH results, different US power levels led to a reduction in the relative fluorescence intensity of aquafaba proteins. It is common for hydrophobic groups to become more exposed after US processing,^38^ which can promote aggregation. As a result, fewer hydrophobic groups remain available to emit fluorescence.

**Figure 2:**
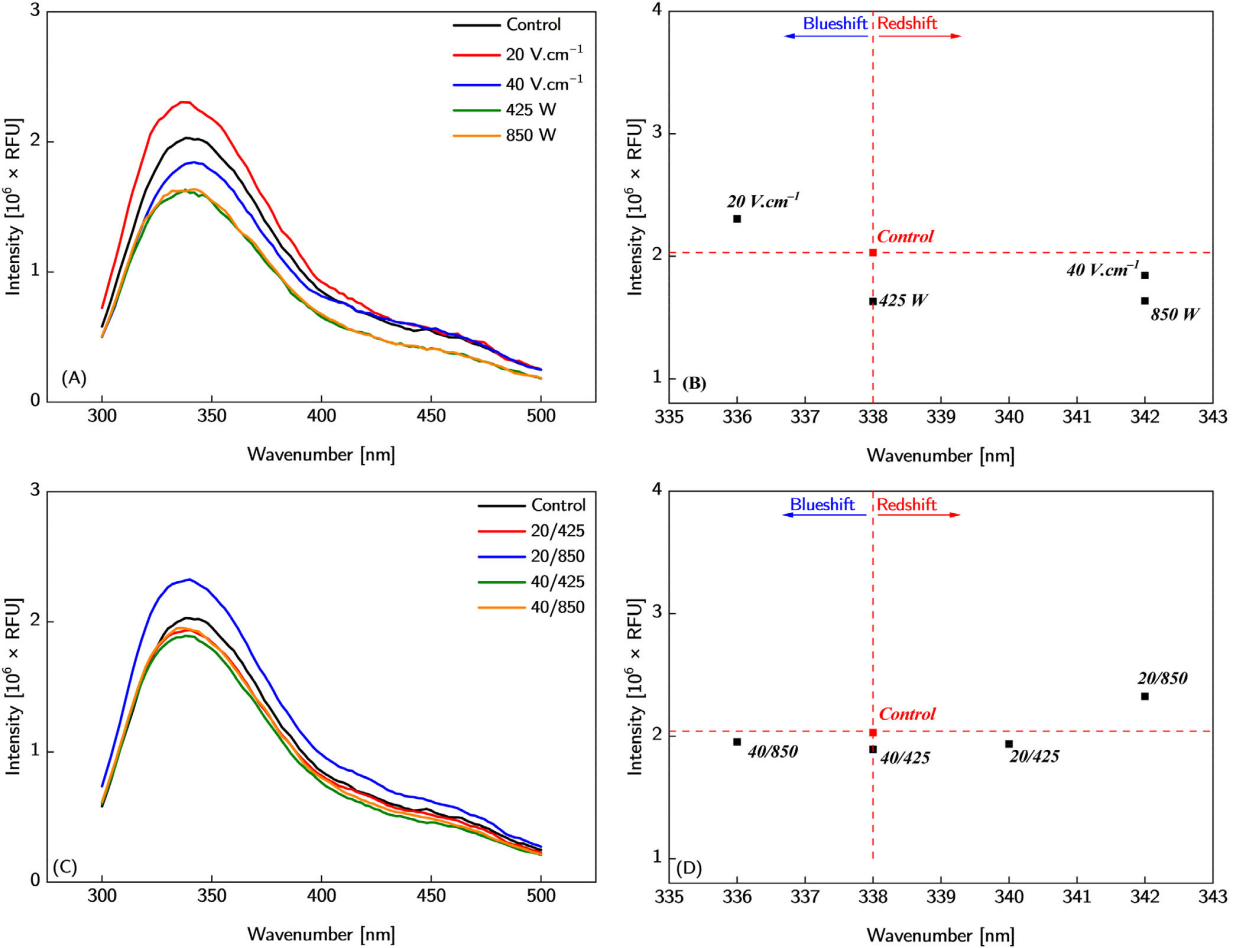
Relative fluorescence and peak shift for aquafaba solutions treated with emerging technologies (ohmic heating and ultrasound) individually (A, B) and combined (C, D). Treatment codes: the first number is the electric field value (20 or 40 V cm^−1^) and the second number is the ultrasound power (425 or 850 W).

Fluorescence spectral shifts were also analyzed to assess how the protein microenvironments were affected by OH and US treatments. These shifts can be classified as *blueshift* or *redshift*, indicating a more hydrophobic or more polar environment surrounding the tryptophan residues, respectively.^39^ At 20 V cm^−1^, a *blueshift* was observed. This behavior is related to the burial of hydrophobic groups within the protein structure as a result of the structural rearrangements induced by OH at this electric field level. When the field intensity increased to 40 V cm^−1^, the maximum fluorescence emission wavelength shifted toward longer wavelengths (*redshift*). This *redshift* corresponds to changes in the hydrophobicity of the protein microenvironment, caused by the displacement of hydrophobic groups (mainly tryptophan) from the protein core toward its surface.^40^ When these hydrophobic residues interact with hydrophilic groups, fluorescence emission decreases, corroborating the relative fluorescence results (Fig. 2(A)).

When combined treatments were applied, OH exerted a more pronounced effect on the structural rearrangement of protein polymer chains. As observed, applying US immediately after OH tended to produce a *redshift* relative to the individual OH treatment (20 V cm^−1^), regardless of US power level. It is possible that the mild OH treatment at lower electric fields induced less extensive structural changes in the proteins. However, once US was applied, the high energy dissipated into the sample further promoted the exposure of hydrophobic groups, enhancing their potential to interact at the oil–water interface, thus improving emulsion formation and stability. Therefore, the combined effect of these technologies proved effective in enhancing protein surface properties that favor emulsion formation (other surface properties such as surface hydrophobicity, free sulfhydryl content, solubility and zeta potential will be discussed in later sections).

At higher electric field intensity combined with US, a *blueshift* tendency was observed. In this case, it appears that the 40 V cm^−1^ field caused extensive structural rearrangement (as shown in Fig. 2(B)) and, following US application, this structure collapsed, re‐embedding the hydrophobic clusters released by OH. It is also possible that the additional energy dissipation during US treatment enhanced hydrophobic–hydrophobic interactions, promoting the formation of aggregates that prevented fluorescence emission.

No previous studies have reported the combined effects of OH and US on aquafaba proteins. However, Shi *et al*.^41^ found no shift in the maximum fluorescence peak of whey protein isolate (WPI) subjected to combined US and high‐pressure (HPH) treatment. Although both US and HPH are known to induce substantial structural modifications in proteins, OH appears to promote milder but more effective modifications, enabling the exposure of active sites of different polarities that can confer essential physicochemical properties for emulsion formation and stability.

### Physical–chemical analysis of treated aquafabas

#### Surface hydrophobicity and free sulfhydryls

**Figure 3:**
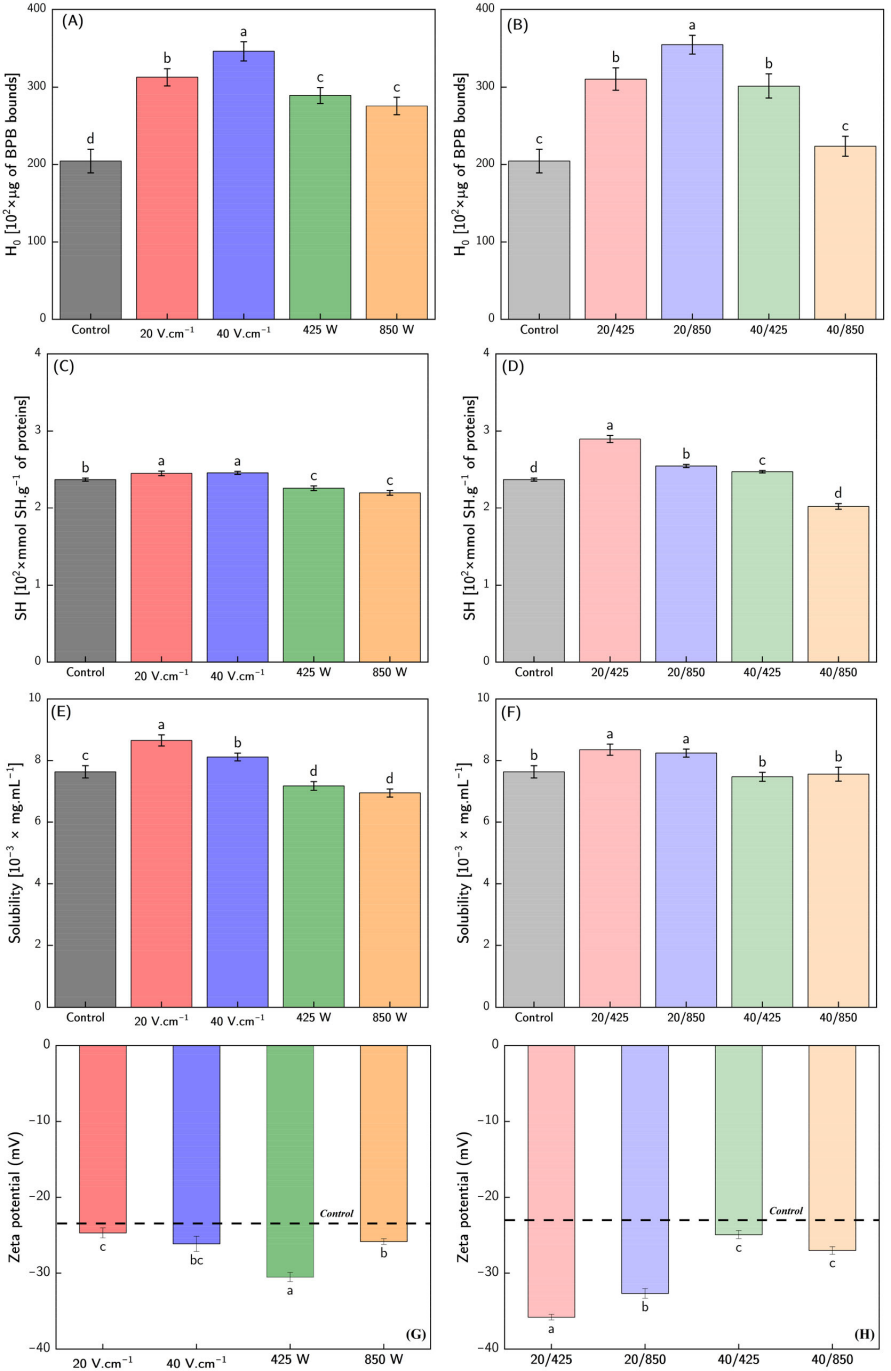
Physicochemical properties of aquafaba modified by emerging technologies (ohmic heating and ultrasound) and their combinations: surface hydrophobicity (A, B), free sulfhydryls (C, D), solubility (E, F) and zeta potential (G, H). The dash pink line is control (untreated aquafaba = −23.43 mV). ^a‐d^ Different letters in the same bar graph mean significant difference by Tukey's test (*P* < 0.05).

The surface hydrophobicity (*H*₀) results for aquafaba proteins modified by individual application of emerging technologies are shown in Fig. 3(A), (B). Different electric field intensities led to significant increases (*P* < 0.05) in *H*₀ compared to the control, with a positive correlation between electric field strength and *H*₀ values. Wang *et al*.^37^ also reported an increase in *H*₀ with increasing electric field intensity during OH modification of soy proteins.

The free sulfhydryl (SH) contents of aquafaba proteins are presented in Fig. 3(C), (D). The control sample exhibited the lowest SH value, as expected for proteins in a more native state, where most cysteine residues are involved in disulfide bonds. In general, although the treatments significantly (*P* < 0.05) increased SH levels, OH treatment alone did not produce markedly high magnitudes of change, unlike the OH treatment of soy proteins, where up to a seven‐fold increase relative to the control was observed.^42^

Ohmic heating at 20 and 40 V cm^−1^ significantly (*P* < 0.05) increased the SH content in AF proteins. This may indicate partial unfolding of proteins and disruption of some disulfide bonds. These structural changes enhance protein surface reactivity for further binding and interactions. Such results are consistent with structural modifications caused by OH. The increase in SH groups is generally beneficial for emulsifying functionality because exposed thiol groups can participate in interfacial crosslinking reactions, strengthening the protein film surrounding oil droplets.^43^, ^44^

Regarding the effect of ultrasound on hydrophobicity, different US power levels did not show significant differences between each other but were higher than the control and lower than OH treatments. Several studies have reported a significant increase in *H*₀ following US processing.^45^, ^46^ The behavior observed here may be explained by (i) the low soluble solids concentration during US processing, which may favor more intense and homogeneous structural modifications,^47^ although such effects could be limited by ultrasound energy diffusion or by the lack of additional hydrophobic sites on the protein molecule; and (ii) the extensive structural changes at higher US power levels, such that newly exposed hydrophobic groups rapidly repolymerize through hydrophobic–hydrophobic interactions^48^ because hydrophobic groups in aqueous environments are thermodynamically unfavorable.^49^

For free sulfhydryl groups (Fig. 3(C)), US exhibited the opposite trend to OH: both 425 and 850 W treatments decreased SH content relative to the control. This effect was more pronounced at 850 W, where the higher acoustic intensity promotes greater free radical generation. Free SH groups are easily oxidized, forming new disulfide bonds.^44^ This suggests that cavitation may have induced thiol oxidation and the formation of new intermolecular disulfide linkages.^50^

Combined treatments produced interesting effects on aquafaba protein *H*₀. The results indicate that OH played a more dominant role in increasing the number of hydrophobic groups on protein surfaces. At a low electric field (20 V cm^−1^), combining OH with US promoted a linear increase in *H*₀ with rising US power. The increase in hydrophobic groups is highly desirable for using proteins as emulsifiers.^51^ However, under higher electric field intensity (40 V cm^−1^), the processes appeared too aggressive, resulting in a reduction of hydrophobic groups to levels similar to the control (40/850 was not significantly different from the control). At high electric fields, protein structures may already be excessively unfolded or denatured, and additional external energy input may favor protein chain repolymerization, re‐embedding hydrophobic groups within the tertiary structure or promoting aggregation.

In the combined 20/425 treatment, SH levels were significantly higher (*P* < 0.05) than in other treatments, showing a 22% increase compared to the control. In this combination, where both temperature and mechanical energy inputs were moderate, little energy was dissipated in forming new disulfide bonds. Consequently, the more flexible protein polymer chains may adsorb more effectively at the oil–water interface,^52^ improving the emulsifying properties of aquafaba proteins.

When SH reduction is excessive (as in 40/850), proteins may form rigid, poorly soluble aggregates, impairing interfacial flexibility and emulsion stability. McClements *et al*.^53^ demonstrated that disulfide bond formation between proteins can promote oil droplet flocculation over time. Therefore, the most favorable condition for emulsions involves controlled SH exposure (as in 20/425), combining greater availability of reactive groups with preserved protein mobility.

#### Protein solubility

Ohmic heating at 20 V cm^−1^ increased AF protein solubility, yielding significantly higher values than the control (*P* < 0.05). This increase is linked to partial protein unfolding, exposing polar groups (Fig. 3(C)) and enhancing water interactions. Treatment at 40 V cm^−1^ also increased solubility compared to the control but to a lesser extent, consistent with previous findings.^14^ As previously reported, higher OH field intensity promotes partial protein aggregation through new disulfide and hydrophobic interactions, reducing protein dispersibility in solution.

Ultrasound at both 425 and 850 W significantly reduced solubility relative to the control (*P* < 0.05) (Fig. 3(E)). Although some studies using US on legume cooking water observed increased protein solubility,^15^, ^16^ the opposite effect observed here may result from the formation of insoluble protein aggregates due to intensified protein–protein interactions. This behavior was also reported by Liu *et al*.,^54^ who observed increased particle size and aggregate formation during ultrasound‐assisted egg white gelation.

Combined treatments 20/425 and 20/850 showed the highest solubility values, both significantly greater than the control (*P* < 0.05) (Fig. 3(F)). These results demonstrate a synergistic effect of both technologies on AF protein solubility: moderate OH treatment promoted initial structural unfolding, and subsequent US application dispersed aggregates, further enhancing solvent interactions. Combined treatments at 40 V cm^−1^ (40/425 and 40/850) did not show significant improvement over the control (*P* > 0.05), indicating that the aggregation induced by the stronger electric field was not reversed by the energy dissipated through US. This finding supports the notion that heavily denatured and crosslinked proteins have limited structural flexibility for redispersion (as also observed for US‐only treatments).

#### Zeta potential

The zeta potential of AF after treatments was measured to assess the effects of OH and US on protein surface charge (Fig. 3(G)). The control aquafaba exhibited a zeta potential of approximately −22 mV. After OH treatment, a slight increase in negative charge was observed, particularly at the lower electric field (20 V cm^−1^). This increase can be attributed to the exposure of carboxylic and sulfonic groups during OH‐induced protein unfolding, which enhances surface charge density and promotes greater electrostatic repulsion between particles.^19^ At 40 V cm^−1^, the negative charge remained close to the control value but was still significantly different (*P* < 0.05). This suggests that stronger electric fields may induce conformational rearrangements and the onset of aggregation,^14^ thereby masking some ionizable surface groups.

US treatments exhibited behavior distinct from OH. At 425 W, the zeta potential became significantly (*P* < 0.05) more negative (−30 mV), indicating that US promotes controlled protein unfolding and exposure of acidic amino acid residues.^55^, ^56^ Additionally, low‐power US can facilitate the disruption of protein aggregates, improving dispersibility.^57^ However, at 850 W, the negative charge slightly decreased (approximately −25 mV). This effect is likely related to oxidation of carboxylate and thiol groups and to aggregate formation via hydrogen and disulfide bonding,^55^ reducing surface charge mobility.

**Figure 4:**
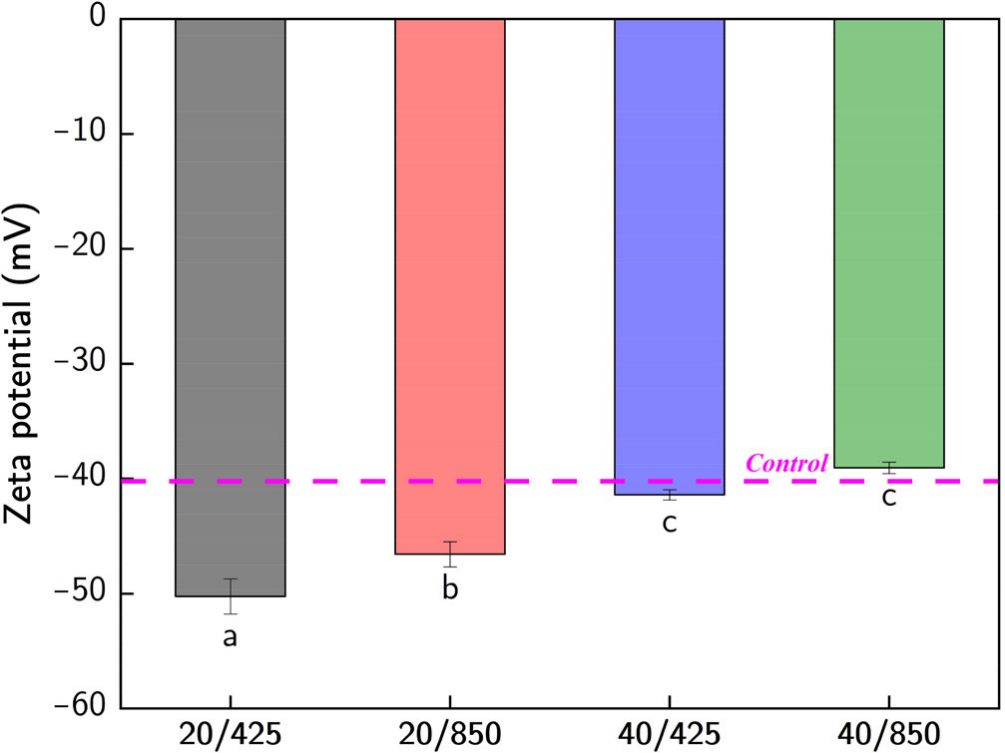
Zeta potential of aquafaba emulsions treated with emerging technologies (ohmic heating and ultrasound) combined. Treatment codes: the first number is the electric field value (20 or 40 V.cm^−1^) and the second number is the ultrasound power (425 or 850 W). The dash pink line is control (aquafaba untreated = −40.24 mV). ^a‐c^ Different letters in the same bar graph mean significant difference by Tukey's test (*P* < 0.05).

Combined treatments exhibited both synergistic and antagonistic effects (Fig. 3(H)). The 20/425 treatment produced the most negative zeta potential among all treatments (−35.79 mV), indicating a highly stable colloidal system with well‐dispersed and strongly charged proteins. This condition promotes molecular repulsion and prevents aggregate formation.^58^ Treatments at 40 V cm^−1^ (40/425 and 40/850) showed less negative values (but still significant, *P* < 0.05), approaching those of the control (Fig. 4). This partial neutralization suggests strong aggregation induced by the higher electric field, which can mask ionizable groups and reduce electrophoretic mobility. The lower availability of free polar groups also reflects reduced protein hydration, consistent with the lower solubility and H₀ values observed in these samples.

Thus, zeta potential results reinforce the role of ohmic heating as the primary driver of structural modification and ultrasound as a fine modulator of colloidal state. The 20/425 combination yielded the most favorable dispersion and electrostatic stability conditions for potential applications in food emulsions. Based on the preceding analyses, we infer that combined OH–US treatments provided the most advantageous physicochemical properties for emulsion formation and stability. The ability to control protein unfolding and aggregation through moderate OH followed by US provides a practical route to design emulsifiers with improved performance, reducing the need for synthetic additives. Consequently, only the combined treatments were considered for the tests and analyses described in the following sections.

### Zeta potential for emulsions

Chickpea aquafabas are primarily composed of proteins, carbohydrates and saponins.^9^, ^11^, ^59^ The distinct chemical natures of these compounds can directly influence the surface charge of this colloidal system. It is well established that proteins exhibit surface charges that are closely related to the stability of emulsion systems.^60^ Starches are the most abundant carbohydrates in aquafaba^11^ and exhibit low surface charge.^61^ Saponins, on the other hand, are amphiphilic compounds characterized by high surface charge,^62^ which plays a key role in stabilizing colloidal systems. To the best of our knowledge, no studies have reported the surface charge values of the saponins present in aquafaba. Therefore, in the discussion of our results, zeta potential values are interpreted as the net sum of the surface charges of aquafaba proteins and saponins.

Ohmic heating (20 and 40 V cm^−1^) did not markedly alter the overall surface charge, maintaining values similar to the control but slightly more negative (Fig. 3(G)). This suggests that partial denaturation exposed some charged groups without causing substantial changes in ionization. These exposed charged residues are likely glutamic and aspartic acid side chains^16^ revealed during structural modification of the proteins. These amino acids carry negative charges at the pH range observed for the aquafaba samples in this study (5.75–6.14).

Ultrasound at 425 W produced the greatest increase in negative surface charge, indicating that cavitation may have exposed acidic groups (carboxyls) and negatively charged amino acid residues, thereby enhancing electrostatic repulsion between particles.^63^ By contrast, the 850 W treatment resulted in a less negative value, closer to that of the control. This suggests that higher acoustic power may have induced protein aggregation and structural reorientation,^64^ reducing the availability of charged surface groups^65^ and, consequently, the ability of proteins to interact effectively at the emulsion interface.^66^

When analyzing the effects of the combined emerging technologies (Fig. 3(H)), the lower electric field intensity promoted a significant (*P* < 0.05) increase in zeta potential compared to the control. The 20/425 treatment exhibited the most negative zeta potential among all samples (approximately −35 mV), indicating extensive exposure of charged groups (Fig. 4). This confirms a synergistic effect between moderate OH and low‐power US: the former induced partial denaturation, whereas the latter dispersed aggregates, maximizing surface charge. The 20/850 treatment also displayed a high negative potential, although slightly lower than 20/425. This may indicate that the higher US energy caused greater oxidation of protein functional groups, leading to minor aggregation.

Conversely, the 40/425 and 40/850 treatments showed less negative values, similar to the control (Fig. 3(H)). This indicates that the stronger electric field promoted protein aggregation, masking charged groups and reducing electrophoretic mobility. Proteins with higher negative surface charge (as in 20/425) are more effective in stabilizing emulsions through electrostatic repulsion between droplets, acting synergistically with the increased solubility and hydrophobicity discussed earlier. By contrast, harsher treatments (40/425 and 40/850) may compromise stability, as aggregates with lower charge densities tend to promote droplet coalescence and colloidal instability.

In summary, the chemical and structural properties of aquafaba proteins indicate the following impacts on emulsion formation: treatments 20/425 and 20/850 led to greater exposure of aromatic groups, increased amphiphilicity, and proteins with enhanced potential to stabilize colloidal systems; whereas treatments 40/425 and 40/850 resulted in greater structural organization and aggregation, reducing key interfacial properties essential for emulsion formation and stability. The effects of these properties on aquafaba emulsions will be discussed in the subsequent sections.

### Rheological analysis

#### Viscosity

**Figure 5:**
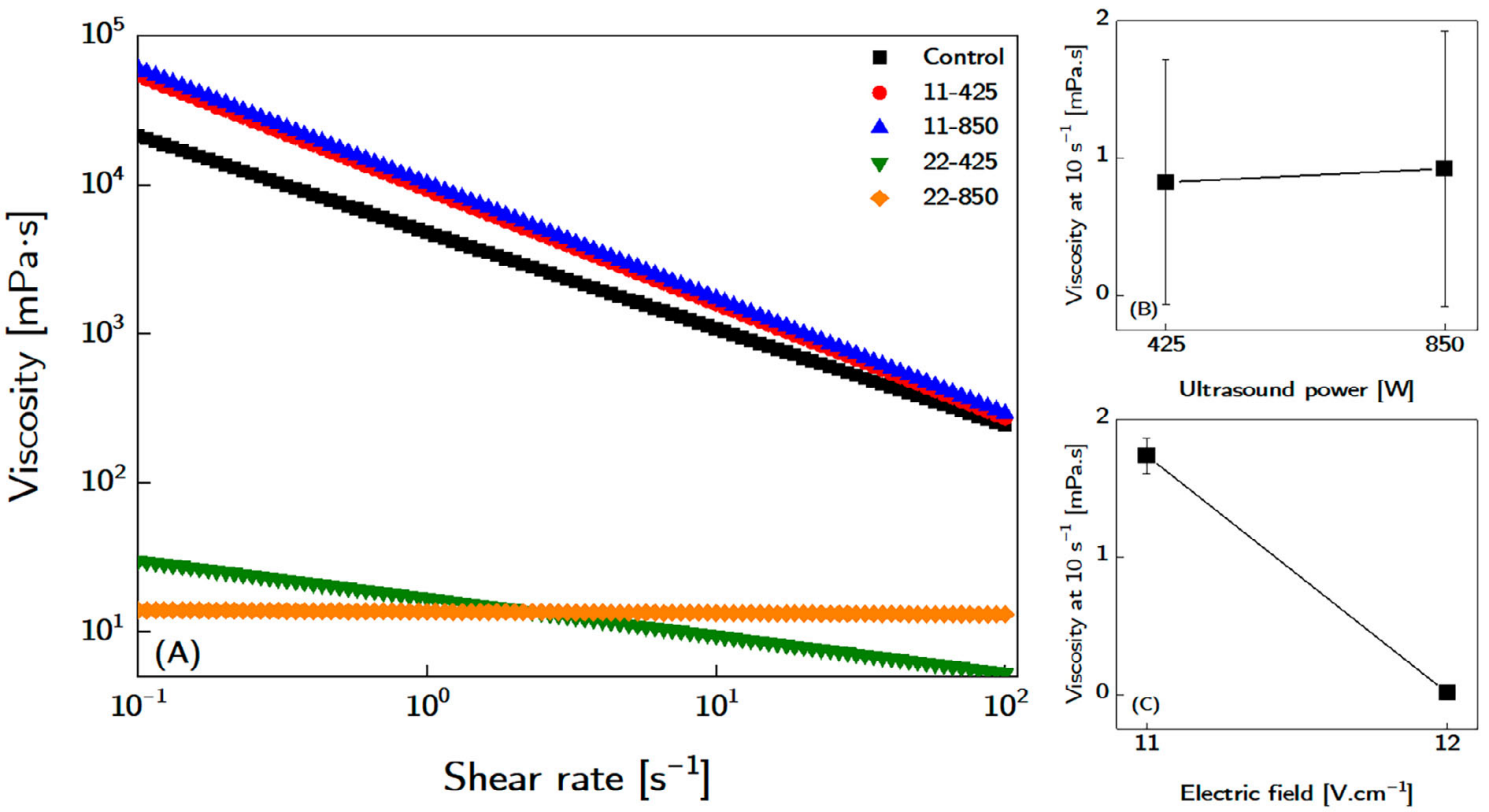
Apparent viscosity (A) and main effects (viscosity at a shear rate of 10 s^−1^) curves of the ultrasonic (B) and ohmic heating (C) modification processes of aquafaba emulsions. Treatment codes: the first number is the electric field value (20 or 40 V cm^−1^) and the second number is the ultrasound power (425 or 850 W). The lines in (B) and (C) are guidelines for the eyes.

The viscosities of the mayonnaise samples are presented in Fig. 5(A). All samples exhibited pseudoplastic (shear‐thinning) behavior, which is typical of complex food systems containing proteins and polysaccharides, such as those found in aquafaba,^67^ where viscosity decreases with increasing shear rate. It was observed that ohmic heating treatments at lower electric field intensities caused an increase in the viscosity of the emulsions. At low electric field levels (20 V cm^−1^), Krichanã *et al*.^14^ reported that ohmic heating increased the hydrophobicity of aquafaba proteins, which may explain our findings. When the hydrophobicity of food proteins increases, their interaction with the lipid phase of emulsions can be strengthened, leading to more cohesive emulsions with higher viscosity.^68^ On the other hand, when hydrophobicity becomes excessive, protein solubility decreases due to the aggregation of polypeptide chains.^69^ As a result, proteins fail to distribute evenly within the continuous phase, producing less uniform and less viscous emulsions, as observed by the reduction in viscosity under the 40 V cm^−1^ electric field.^70^ Furtado *et al*.^71^ also observed that emulsions containing lactoferrin treated by ohmic heating exhibited a four‐fold increase in viscosity compared to emulsions prepared with untreated proteins. The different ultrasound power levels tested did not show any significant effect (*P* > 0.05).

The shear stress data as a function of the shear rate of the emulsions were fitted to the Power Law model (Table 2). The parameter *n* represents the flow behavior of fluids, which can be Newtonian (*n* = 1), pseudoplastic (*n* < 1) or dilatant (*n* > 1).^72^ The results confirm the effects previously observed: emulsions treated at 20 V cm^−1^ were thicker, more pseudoplastic, and more stable under shear (higher *k* values and lower *n* values), whereas samples treated at higher electric field intensity (40 V cm^−1^) behaved as more fluid, less structured systems, exhibiting flow behavior closer to Newtonian (a sharp decrease in *k* and an increase in *n*).

**Table 2:** Consistency index (*k*), flow behavior index (*n*) and determination coefficient (*R* ^2^) of aquafaba emulsions

Assays	*K*	*n*	*R* ^2^
Control	3224 ± 1.200 b	0.334 ± 0.001 b	0.999
20/425	9214 ± 3.700 a	0.234 ± 0.001 b	0.999
20/850	10 220 ± 4.100 a	0.228 ± 0.002 b	0.998
40/425	16.83 ± 0.010 c	0.748 ± 0.001 a	0.999
40/850	13.68 ± 0.010 c	0.990 ± 0.002 a	0.999

Values are expressed as the mean ± SD (*n* = 3). Different lowercase letters within the same column indicate significant differences (*P* < 0.05). Treatment codes: the first number refers to the electric field strength (20 or 40 V cm^−1^) and the second to the ultrasound power (425 or 850 W).

#### Frequency sweep

During a frequency sweep, the oscillation angular frequency (*ω*) varies, whereas the strain is kept constant. This test determines the structural response over different experimental times: a relatively low *ω*, such as 1 Hz, can be considered a long time (1 s), whereas a relatively high *ω*, such as 100 Hz, corresponds to a short time (0.01 s).^73^

**Figure 6:**
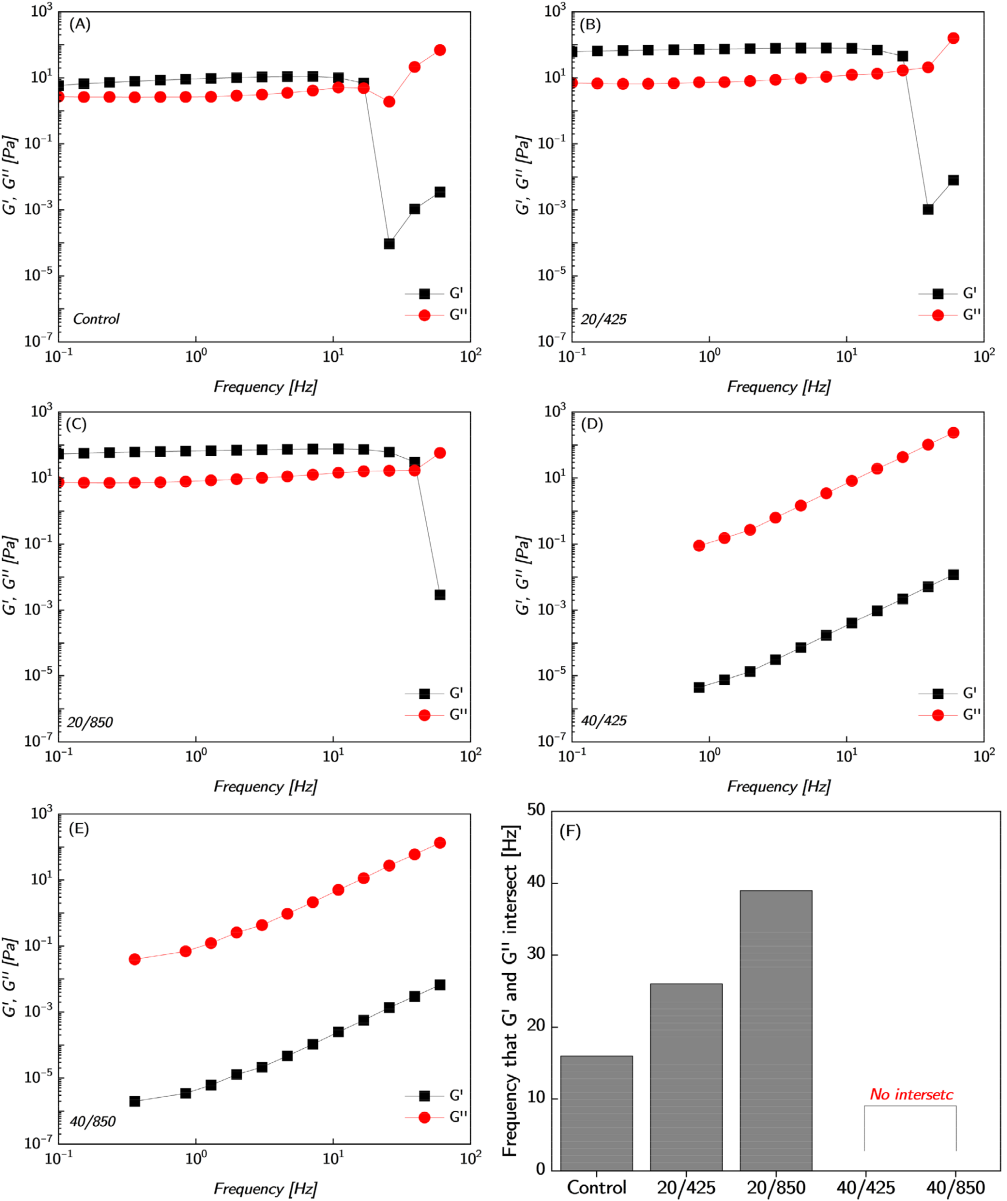
Frequency sweep of aquafaba emulsions treated with combined OH and US (A–E) and the frequencies where *G*′ and *G*″ intersect (F). Treatment codes: the first number refers to the electric field strength (20 or 40 V cm^−1^) and the second to the ultrasound power (425 or 850 W). Lines are guides for the eye.

The frequency sweep results for the aquafaba emulsions are shown in Fig. 6(A)–(E). The control emulsion exhibited *G*′ > *G*″ over most of the frequency range, with a crossover point around 16 Hz. This indicates that the control emulsion possesses a predominantly elastic character, typical of a relatively well‐organized structural network. When the effects of the treatments were analyzed, emulsions treated with high electric field intensity (40 V cm^−1^) showed markedly different behavior compared to those treated under low electric field intensity (20 V cm^−1^). At 20 V cm^−1^, *G*′ remained greater than *G*″ across almost the entire frequency range, with *G*′ values up to 10 times higher than those of the control emulsion.

The crossover frequencies were also higher than those of the control (26 and 39 Hz for 20/425 and 20/850, respectively) (Fig. 6(F)). This suggests that ohmic heating at lower electric field intensities induced partial protein denaturation, enhancing interfacial adsorption and the mechanical strength of the aquafaba emulsions. The reduction in *G*′ at high frequencies may be associated with the partial disruption of weak interactions (hydrogen bonds and hydrophobic interactions) under increased mechanical stress.^74^ Higher ultrasound power also improved the mechanical resistance of the aquafaba emulsions, shifting the crossover between *G*′ and *G*″ to higher frequencies compared to the control. The increased energy transfer from ultrasound to aquafaba may have enhanced the interfacial properties of macromolecules, improving oil–protein interactions and consequently promoting a viscoelastic structure similar to gels.^75^

At high electric field intensity (40 V cm^−1^), however, an inversion occurred: *G*″ became higher than *G*′ throughout the entire frequency range, indicating a predominantly viscous system. Both elastic and viscous moduli showed frequency dependence across the range tested, consistent with a weaker gel or a less stable emulsion.^76^ The higher electric field intensity likely induced excessive protein denaturation and the formation of insoluble aggregates, preventing the establishment of an efficient elastic network and impairing structural resistance under stress. Furthermore, higher ultrasound power further weakened and destabilized the polymeric network.

### Multiple light scattering

**Figure 7:**
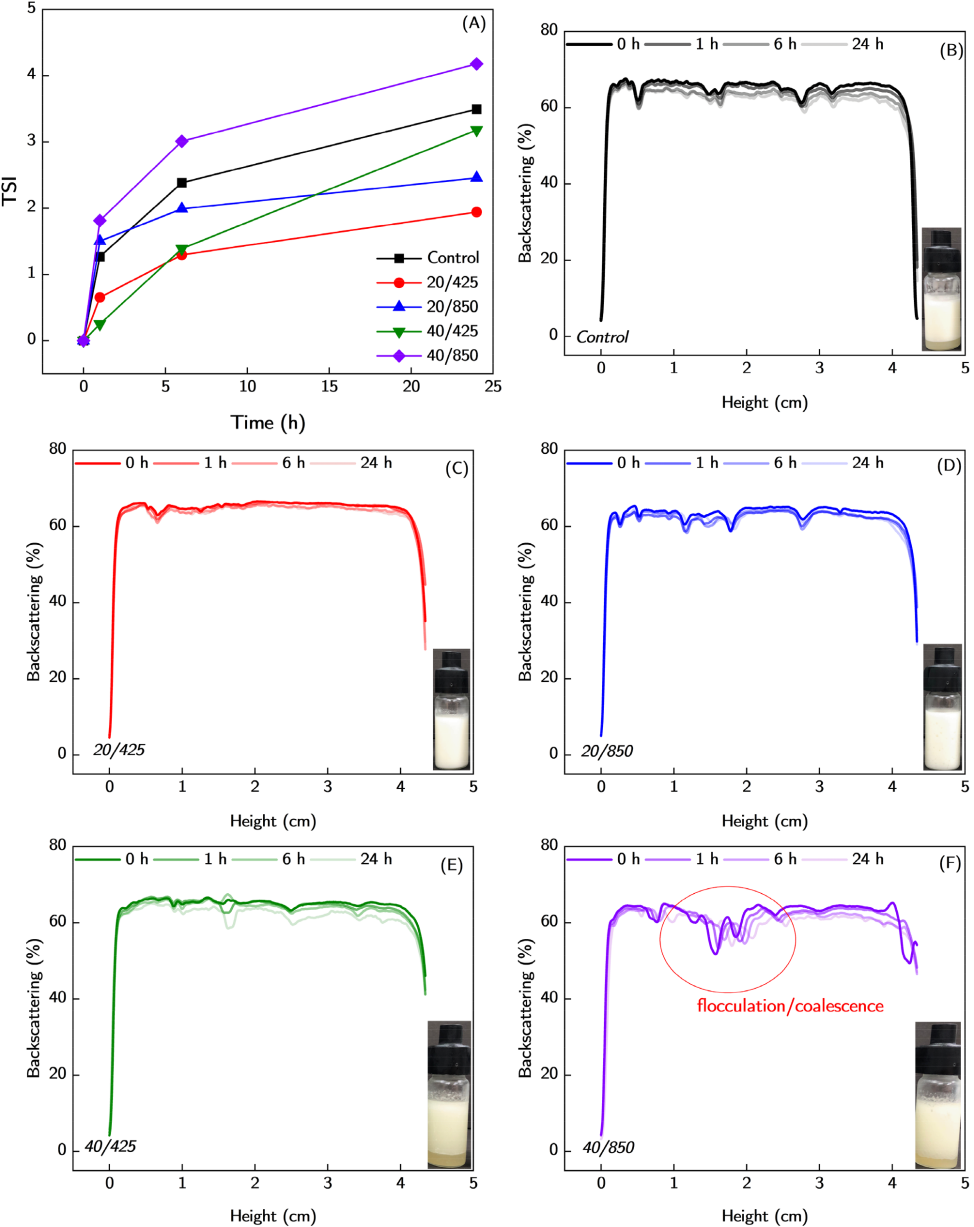
Turbiscan stability index (TSI) (A) and backscattering profiles (B–F) for aquafaba emulsions modified by combined ohmic heating and ultrasound treatments. The visual appearance of emulsions after 24 h is shown. Treatments are coded as follows: the first number refers to the electric field strength (20 or 40 V cm^−1^) and the second to the ultrasound power (425 or 850 W).

Colloidal stability depends on a delicate balance: controlled denaturation increases interfacial activity, whereas excessive energy input, whether from a strong electric field or high ultrasound power, leads to the formation of insoluble aggregates that accelerate destabilization processes. The aquafaba emulsions demonstrated good physical stability, with TSI values below 4 after 24 h of analysis (Fig. 7(A)). Raikos *et al*.^77^ also reported TSI values below 4 regardless of oil concentration (70–80%). By contrast, much higher values were observed for emulsions containing myofibrillar proteins combined with soy, corn, sunflower, flaxseed and rapeseed proteins, which reached TSI values greater than 10 within 2 h of observation.^66^ The protein‐, saponin‐ and amphiphilic glycoside‐rich composition of aquafaba contributes to its high emulsifying capacity.^78^

Among the treatments, only sample 40/850 showed reduced stability compared with the control over time. In 40/850, flocculation was more evident (highlighted in Fig. 7(F)), likely related to abrupt structural modifications in proteins caused by high electric field intensities during ohmic heating^14^ and by ultrasound power.^79^ When aquafaba was treated under a lower electric field, the emulsions exhibited greater stability. These results are consistent with the hydrophobicity data (Fig. 3(A), (B)) because the 20 V cm^−1^ treatment promoted exposure of hydrophobic groups that enhanced protein–lipid interactions within the emulsions.

The backscattering (BS) results provide insights into destabilization phenomena occurring under the influence of gravity alone. Only minimal destabilization events were observed during storage (Fig. 7(B)–(E)). No creaming or sedimentation was detected for the low electric field treatments (20 V cm^−1^) during 24 h of analysis. This agrees with the viscosity data, which showed that low‐field ohmic heating produced more viscous emulsions. These findings are linked to structural changes in proteins during processing, resulting in conformations more favorable for interaction with the lipid phase and, consequently, higher viscosity.

In other treatments, in addition to slight creaming, variations in BS were observed in the central portion of the emulsions, possibly associated with the growth of oil droplets (flocculation or coalescence).^80^ When higher ultrasound power (850 W) and electric field intensity (40 V cm^−1^) were applied, more pronounced agglomeration/coalescence occurred in the middle region of the vials containing the emulsions (the central TSI values of the 40/850 emulsions were the highest among all treatments; see Supporting information, Fig. S1).

Using a less energetic ultrasonic bath (maximum power 280 W), Noh and Lee^16^ reported that the emulsifying properties of chickpea aquafaba improved significantly compared with the control and continued to improve with longer processing times. These findings reinforce that, in the present study, the application of high‐energy ultrasound caused excessive structural modification of proteins, mainly aggregation, thereby reducing emulsifying properties.

By contrast to our results, Shi *et al*.^41^ found that the synergistic combination of ultrasound with another technology (high pressure) enhanced the emulsifying properties of WPI. However, Shi *et al*.^41^ used ultrasound powers up to 600 W, which is lower than the 850 W applied in the present study. It can therefore be inferred that, in addition to the inherently superior emulsifying properties of WPI, the high ultrasound power used here may have caused the emulsifying biopolymers in aquafaba to lose their interfacial functionality, reducing emulsion stability. These findings are particularly relevant for plant‐based formulations, where achieving stable emulsions with clean‐label ingredients remains a major technological challenge.

### Confocal microscopy

**Figure 8:**
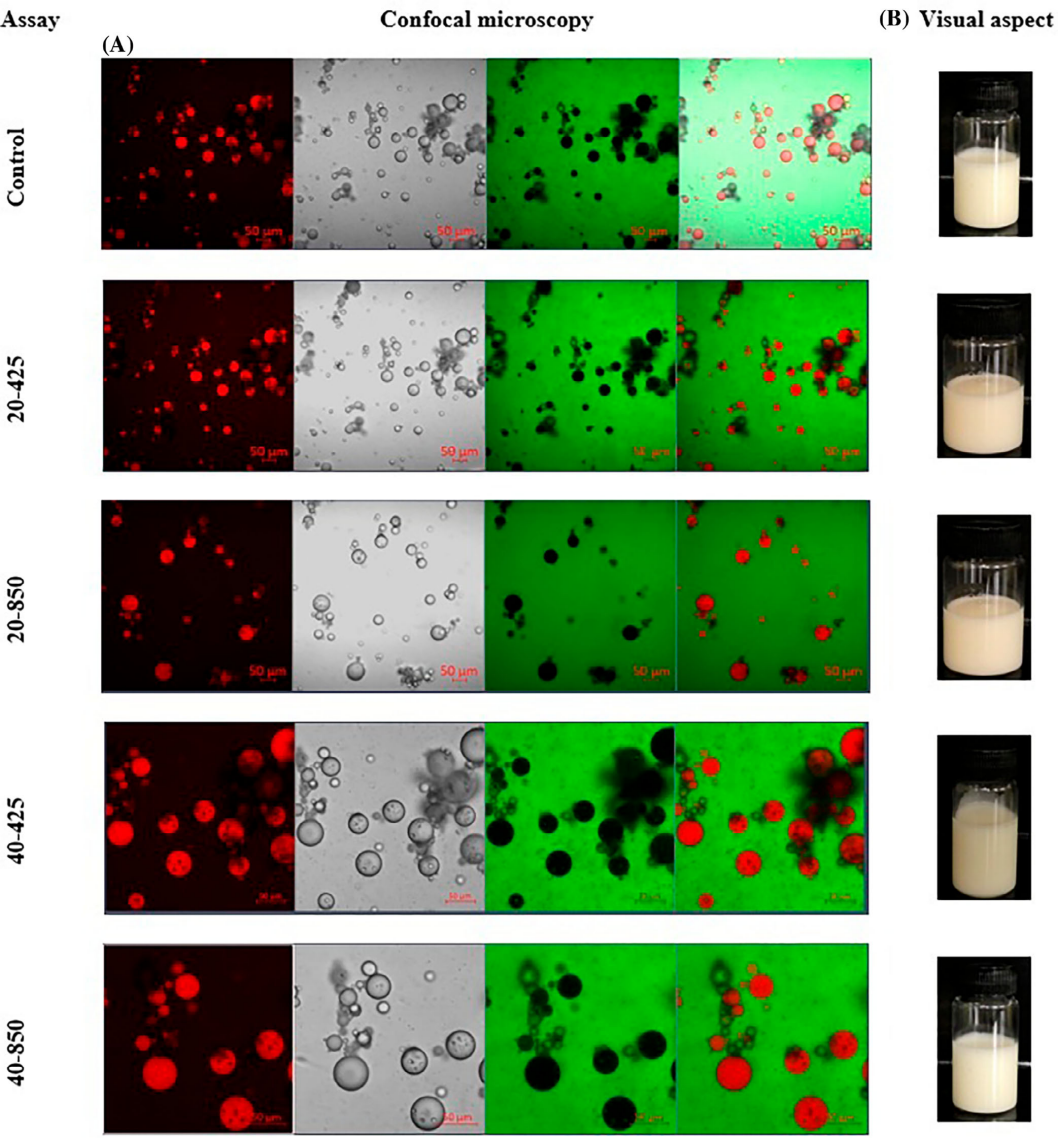
Confocal micrographs (A) and visual appearance (B) of emulsions prepared with aquafabas modified by combined ohmic heating and ultrasound. Treatment codes: the first number represents the electric field intensity (20 or 40 V cm^−1^) and the second number represents the ultrasound power (425 or 850 W).

Confocal microscopy images and the visual appearance of the emulsions (Fig. 8) support the findings obtained from the chemical and techno‐functional characterization of aquafabas modified by combined ohmic heating and ultrasound. In the control sample, the droplets displayed a heterogeneous distribution with few signs of coalescence. Treatments at 20 V cm^−1^, particularly 20/425, produced emulsions with smaller, more homogeneous droplets that were well coated with proteins, resulting in white, opaque and stable emulsions. By contrast, at 40 V cm^−1^, larger, polydisperse and poorly coated droplets were observed, leading to translucent and unstable emulsions. Wang *et al*.^81^ similarly reported that increasing electric field intensity during ohmic heating promoted aggregation in soy proteins. Wang *et al*.^81^ noted that when the electric field intensity exceeds a certain threshold, proteins rapidly aggregate, resulting in the formation of insoluble protein aggregates.

## CONCLUSIONS

The results of our study demonstrate that OH was the primary determinant of structural modifications, whereas US acted as a modulator of molecular arrangements in aquafaba proteins. The application of a moderate electric field (20 V cm^−1^), particularly in combination with low‐power ultrasound (425 W), produced synergistic effects by enhancing solubility, hydrophobicity, sulfhydryl exposure and electrostatic stabilization, which collectively improved the rheology, stability, and microstructure of the emulsions. Although higher electric field intensities (≥ 40 V cm^−1^) induced excessive denaturation and aggregation of aquafaba protein chains, thereby reducing solubility and interfacial activity, the combination with US showed that these negative effects were partially mitigated. Acoustic cavitation promoted the redistribution of aggregates formed during ohmic heating and re‐exposed functional groups (hydrophobic and sulfhydryl), restoring protein flexibility and increasing emulsion stability. Thus, OH primarily induces structural unfolding and controlled denaturation, whereas US finely tunes these structural changes, allowing even less favorable electric field conditions to yield enhanced techno‐functional performance. OH defines the structure of the proteins, whereas US adjusts the arrangement and dispersion of that structure. This study provides mechanistic insights into the structure–function relationships of aquafaba proteins and supports the rational design of plant‐based emulsions with tailored stability and texture.

## AUTHOR CONTRIBUTIONS

All authors participated in the conception and design of the study, the analysis and interpretation of data, and drafting the article or revising it critically for important intellectual content. All authors approved the final version of the manuscript submitted for publication.

## CONFLICTS OF INTEREST

The authors declare that they have no conflicts of interest.

## Supporting information


**Figure S1.** Turbiscan stability index (TSI) for the middle portion of aquafaba emulsions modified by ohmic heating and ultrasound. Treatment codes: the first number represents the electric field strength (20 or 40 V cm^−1^) and the second number represents the ultrasound power (425 or 850 W). The lines are guidelines for the eyes.

## Data Availability

The data that support the findings of this study are available from the corresponding author upon reasonable request.
