# Supplementary material for: Ohmic heating dominates and ultrasound modulates aquafaba structure: A combined approach to design stable plant‐based emulsions
Source: J Sci Food Agric. 2026 May 27;106(12):7109–24. doi: 10.1002/jsfa.70743 (PMC13442761; doi:10.1002/jsfa.70743)
Supplement: Supplementary file 1 [file JSFA-106-7109-s001.docx]

**Supplementary material**

**Ohmic heating dominates and ultrasound modulates aquafaba structure: a combined approach to design stable plant-based emulsions**

Débora Krichanã^1,2^, Thais Caroline Buttow Rigolon^1,2^, Paula Zambe Azevedo^1,2^, João Paulo Lins^1,2^, Gabriel Oliveira Horta^1,2^, Linamarys Aparecida de Oliveira Paulo^3^, Márcia Cristina Teixeira Ribeiro Vidigal^3^, Taline Amorim Santos^4^, Isis Rodrigues Toledo Renhe^4^, Jaqueline de Araújo Bezerra^5^, Paulo César Stringheta^1,2^, Evandro Martins^1,2*^, Pedro Henrique Campelo^1,2*^

^1^LaCBio, Laboratory of Natural Pigments and Bioactives, Food Technology Department, Federal University of Viçosa, Viçosa 35570-900, Brazil.

^2^LHMA, Laboratory of Hygiene and Food Microbiology, Food Technology Department, Federal University of Viçosa, Viçosa 35570-900, Brazil.

^3^Food Technology Department, Federal University of Viçosa, 36570-900 Viçosa, Brazil

^4^ EPAMIG - Instituto de Laticínios Cândido Tostes, 36045-560, Brazil

^5^ Federal Institute of Education, Science, and Technology of Amazonas, Manaus, Brazil

*Corresponding author: [evandromartins@ufv.br](mailto:evandromartins@ufv.br) (E. Martins); [pedrocampelo@ufv.br](mailto:pedrocampelo@ufv.br) (P. H. Campelo)

Figure S1 – Turbiscan stability index (TSI) for the middle portion of aquafaba emulsions modified by ohmic heating and ultrasound. The treatments are coded as follows: the first number represents the electric field strength (20 or 40 V·cm−1), and the second number represents the ultrasound power (425 or 850 W). The lines are guides for the eyes.
